# 3-(3,4-Dihydroxyphenyl)-1-methoxy-1-oxopropan-2-aminium chloride

**DOI:** 10.1107/S160053681201728X

**Published:** 2012-04-25

**Authors:** Ze-Feng Hou, Juan Feng, Jing-Tian Liu, Xiao-Li Zhen, Jian-Rong han han

**Affiliations:** a College of Sciences, Hebei University of Science & Technology, Shijiazhuang 050018, People’s Republic of China; b College of Chemical & Pharmaceutical Engineering, Hebei University of Science & Technology, Shijiazhuang 050018, People’s Republic of China; c College of Chemistry and Chemical Engineering, Nanjing University of Technology, Nanjing 211816, People’s Republic of China

## Abstract

In the title compound, C_10_H_14_NO_4_^+^·Cl^−^, the benzene ring makes a dihedral angle of 64.68 (4)° with the methyl­amino­propano­ate unit, which is bonded to the catechol ring *via* a methyl­ene C atom. A strong intra­molecular O—H⋯O hydrogen bond occurs. In the crystal, O—H⋯O, N—H⋯Cl and O—H⋯Cl hydrogen bonds and weak C—H⋯O inter­actions link the mol­ecules into a three-dimensional network.

## Related literature

For medicinal applications of the title compound, see: Cooper *et al.* (1984 ▶). For a related structure, see: Naicker *et al.* (2012 ▶)
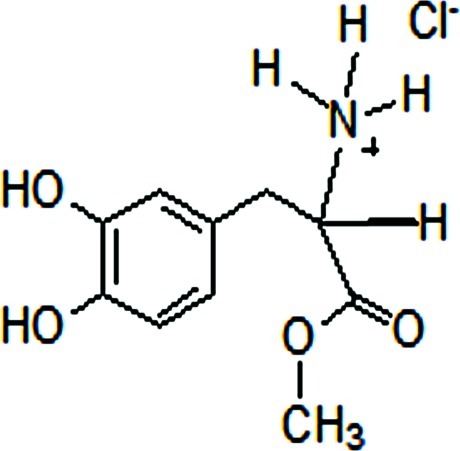


## Experimental

### 

#### Crystal data

C_10_H_14_NO_4_^+^·Cl^−^
*M* *_r_* = 247.67
Orthorhombic, 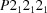

*a* = 4.9969 (15) Å
*b* = 14.498 (4) Å
*c* = 16.109 (5) Å
*V* = 1167.1 (6) Å^3^
*Z* = 4
Mo *K*α radiation
μ = 0.33 mm^−1^
*T* = 113 K
0.20 × 0.18 × 0.12 mm

#### Data collection

Rigaku Saturn724 CCD diffractometer
Absorption correction: multi-scan (*CrystalClear*; Rigaku/MSC, 2005 ▶) *T*_min_ = 0.938, *T*_max_ = 0.962
12293 measured reflections
2796 independent reflections
2054 reflections with *I* > 2σ(*I*)
*R*_int_ = 0.051

#### Refinement

*R*[*F* ^2^ > 2σ(*F* ^2^)] = 0.027
*wR*(*F* ^2^) = 0.061
*S* = 0.93
2796 reflections
164 parameters
H atoms treated by a mixture of independent and constrained refinement
Δρ_max_ = 0.18 e Å^−3^
Δρ_min_ = −0.23 e Å^−3^
Absolute structure: Flack (1983 ▶), 1117 Friedel pairs
Flack parameter: −0.03 (5)

### 

Data collection: *CrystalClear* (Rigaku/MSC, 2005 ▶); cell refinement: *CrystalClear*; data reduction: *CrystalClear*; program(s) used to solve structure: *SHELXS97* (Sheldrick, 2008 ▶); program(s) used to refine structure: *SHELXL97* (Sheldrick, 2008 ▶); molecular graphics: *SHELXTL* (Sheldrick, 2008 ▶); software used to prepare material for publication: *SHELXTL*.

## Supplementary Material

Crystal structure: contains datablock(s) I, global. DOI: 10.1107/S160053681201728X/pv2536sup1.cif

Structure factors: contains datablock(s) I. DOI: 10.1107/S160053681201728X/pv2536Isup2.hkl

Supplementary material file. DOI: 10.1107/S160053681201728X/pv2536Isup3.cml

Additional supplementary materials:  crystallographic information; 3D view; checkCIF report

## Figures and Tables

**Table 1:** Hydrogen-bond geometry (Å, °)

*D*—H⋯*A*	*D*—H	H⋯*A*	*D*⋯*A*	*D*—H⋯*A*
N1—H1*C*⋯Cl1^i^	0.96 (2)	2.29 (2)	3.209 (2)	161 (2)
N1—H1*B*⋯Cl1	0.92 (2)	2.49 (2)	3.178 (2)	132 (1)
N1—H1*A*⋯Cl1^ii^	1.01 (2)	2.13 (2)	3.112 (2)	163 (2)
O2—H2⋯Cl1^iii^	0.84 (2)	2.26 (2)	3.086 (2)	167 (2)
O1—H1⋯O2	0.83 (2)	2.27 (2)	2.698 (2)	113 (2)
O1—H1⋯O3^iv^	0.83 (2)	2.26 (2)	3.029 (2)	155 (2)
C8—H8⋯O3^ii^	1.00	2.44	3.300 (2)	144
C10—H10*C*⋯O1^v^	0.98	2.47	3.137 (3)	125

Symmetry codes: (i) 
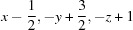
; (ii) 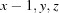; (iii) 
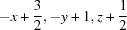
; (iv) 
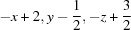
; (v) 
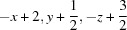
.

**Fig. 1.**
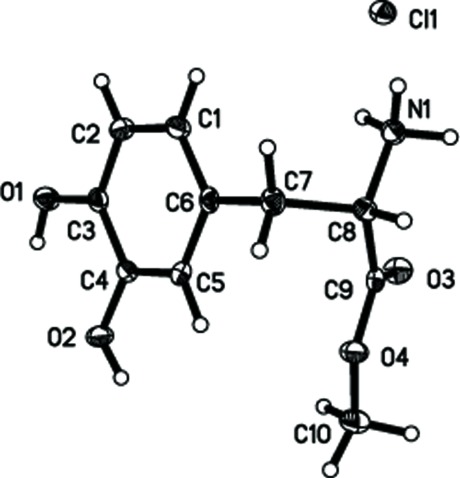
The molecular structure of the title compound with the atom numbering scheme. Displacement ellipsoids are drawn at the 30% probability level. H atoms are presented as small spheres of arbitrary radius.

**Fig. 2.**
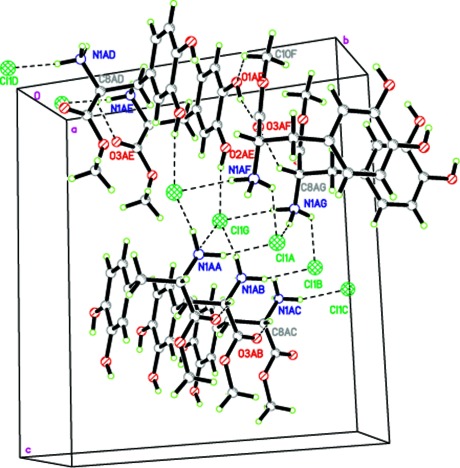
A view of the unit cell showing partial packing of the title compound; hydrogen bonds are shown as dotted lines.

## supplementary crystallographic information

### Comment

A systemic or subcutaneous infusion of *L*-Dopa methyl ester to patients suffering from Parkinson's disease who experience response fluctuations may provide a means of maintaining mobility (Cooper *et al.*, 1984). In this article, we report the synthesis and crystal strucure of *L*-Dopa methyl ester hydrochloride.

In the title compound (Fig. 1), benzene ring makes a dihedral angle of 64.68 (4)° with the methylaminopropanoate moiety (N1/C8–C10/O3/O4) bonded to the catechol ring *via* a methylene C7 atom. The torsion angles in the chain connected with the aromatic ring (C6/C7—C8/N1) and (C6/C7—C8/C9) are 71.39 (19) and -50.5 (2)°, respectively. The crystal packing is stabilized by strong intermolecular O—H···O, N—H···Cl and O—H···Cl hydrogen bonds and further consolidated by weak C—H···O interactions; all the interactions link the molecules into an infinite network (Table 1 and Fig. 2).

The bond distances and bond angles in the title compound are in agreement with the corresponding bond legths and bond angles reported in the crystal structure of a closely related compound (Naicker *et al.*, 2012).

### Experimental

SOCl_2_ (10 ml) was added into MeOH (60 ml) in a reaction flake at 273 K and *L*-3,4-dihydroxyphenylalanine (5.0 g, 25 mmol) was gradually added to this mixture. The temprature was increased to room temperature. After 24 h. the solvent was removed in Vacuo, to yield a white solid. The solid product was recrystallized from ethyl acetate by slow evaporation in the form of colorless single crystals of the title compound suitable for X-ray analysis.

### Refinement

An absolute structure was determined by the Flack (1983) method using 1117 Friedel pairs of reflections which were not merged. The H atoms bonded to N and O-atoms were located from a difference Fourier map and were allowed to refine freely. The H atoms bonded to C-atoms were positioned geometrically and refined using in riding mode, with C—H = 0.95, 0.98, 0.99 and 1.00 Å, for aryl, methyl, methylene and methyne H-atoms, respectively; the *U*_iso_(H) were allowed at 1.5*U*_eq_(C methyl) or 1.2*U*_eq_(C non-methyl).

### Crystal data

**Table:**

C_10_H_14_NO_4_^+^·Cl^−^	*F*(000) = 520
*M**_r_* = 247.67	*D*_x_ = 1.410 Mg m^−^^3^
Orthorhombic, *P*2_1_2_1_2_1_	Mo *K*α radiation, λ = 0.71073 Å
Hall symbol: P 2ac 2ab	Cell parameters from 3847 reflections
*a* = 4.9969 (15) Å	θ = 1.9–27.9°
*b* = 14.498 (4) Å	µ = 0.33 mm^−^^1^
*c* = 16.109 (5) Å	*T* = 113 K
*V* = 1167.1 (6) Å^3^	Prism, colourless
*Z* = 4	0.20 × 0.18 × 0.12 mm

### Data collection

**Table:**

Rigaku Saturn724 CCD diffractometer	2796 independent reflections
Radiation source: rotating anode	2054 reflections with *I* > 2σ(*I*)
Multilayer monochromator	*R*_int_ = 0.051
Detector resolution: 14.22 pixels mm^-1^	θ_max_ = 27.9°, θ_min_ = 1.9°
ω and φ scans	*h* = −6→6
Absorption correction: multi-scan (*CrystalClear*; Rigaku/MSC, 2005)	*k* = −18→19
*T*_min_ = 0.938, *T*_max_ = 0.962	*l* = −21→21
12293 measured reflections	

### Refinement

**Table:**

Refinement on *F*^2^	Secondary atom site location: difference Fourier map
Least-squares matrix: full	Hydrogen site location: inferred from neighbouring sites
*R*[*F*^2^ > 2σ(*F*^2^)] = 0.027	H atoms treated by a mixture of independent and constrained refinement
*wR*(*F*^2^) = 0.061	*w* = 1/[σ^2^(*F*_o_^2^) + (0.0218*P*)^2^] where *P* = (*F*_o_^2^ + 2*F*_c_^2^)/3
*S* = 0.93	(Δ/σ)_max_ = 0.001
2796 reflections	Δρ_max_ = 0.18 e Å^−^^3^
164 parameters	Δρ_min_ = −0.23 e Å^−^^3^
0 restraints	Absolute structure: Flack (1983), 1117 Friedel pairs
Primary atom site location: structure-invariant direct methods	Flack parameter: −0.03 (5)

### Special details

**Table:**

Geometry. All e.s.d.'s (except the e.s.d. in the dihedral angle between two l.s. planes) are estimated using the full covariance matrix. The cell e.s.d.'s are taken into account individually in the estimation of e.s.d.'s in distances, angles and torsion angles; correlations between e.s.d.'s in cell parameters are only used when they are defined by crystal symmetry. An approximate (isotropic) treatment of cell e.s.d.'s is used for estimating e.s.d.'s involving l.s. planes.
Refinement. Refinement of *F*^2^ against ALL reflections. The weighted *R*-factor *wR* and goodness of fit *S* are based on *F*^2^, conventional *R*-factors *R* are based on *F*, with *F* set to zero for negative *F*^2^. The threshold expression of *F*^2^ > σ(*F*^2^) is used only for calculating *R*-factors(gt) *etc*. and is not relevant to the choice of reflections for refinement. *R*-factors based on *F*^2^ are statistically about twice as large as those based on *F*, and *R*- factors based on ALL data will be even larger.

### Fractional atomic coordinates and isotropic or equivalent isotropic displacement parameters (Å^2^)

**Table:**

	*x*	*y*	*z*	*U*_iso_*/*U*_eq_	
O1	1.0416 (3)	0.23597 (8)	0.63923 (7)	0.0197 (3)	
H1	1.079 (4)	0.2226 (12)	0.6879 (12)	0.029*	
O2	0.8286 (3)	0.30070 (8)	0.78169 (8)	0.0217 (3)	
H2	0.756 (4)	0.3261 (12)	0.8230 (13)	0.033*	
O3	0.6921 (2)	0.64877 (8)	0.71119 (8)	0.0195 (3)	
O4	0.3362 (2)	0.59134 (8)	0.77989 (8)	0.0178 (3)	
N1	0.4423 (3)	0.63903 (10)	0.56253 (9)	0.0161 (3)	
C1	0.5531 (3)	0.39895 (10)	0.55641 (11)	0.0181 (3)	
H1D	0.4956	0.4210	0.5038	0.022*	
C2	0.7537 (3)	0.33268 (10)	0.56033 (12)	0.0179 (4)	
H2A	0.8285	0.3086	0.5106	0.022*	
C3	0.8447 (3)	0.30165 (11)	0.63657 (11)	0.0147 (4)	
C4	0.7284 (3)	0.33587 (10)	0.70898 (11)	0.0148 (4)	
C5	0.5249 (3)	0.39978 (10)	0.70502 (11)	0.0162 (4)	
H5	0.4443	0.4213	0.7548	0.019*	
C6	0.4353 (3)	0.43341 (10)	0.62813 (10)	0.0146 (4)	
C7	0.2087 (3)	0.50298 (11)	0.62467 (11)	0.0171 (4)	
H7A	0.0765	0.4871	0.6681	0.020*	
H7B	0.1181	0.4972	0.5703	0.020*	
C8	0.2934 (3)	0.60417 (11)	0.63659 (11)	0.0140 (4)	
H8	0.1278	0.6422	0.6433	0.017*	
C9	0.4670 (3)	0.61855 (10)	0.71259 (10)	0.0143 (3)	
C10	0.4845 (4)	0.59408 (12)	0.85779 (10)	0.0236 (4)	
H10A	0.6548	0.5614	0.8511	0.035*	
H10B	0.3790	0.5644	0.9015	0.035*	
H10C	0.5195	0.6584	0.8731	0.035*	
H1A	0.310 (4)	0.6414 (16)	0.5153 (14)	0.058 (7)*	
H1B	0.598 (3)	0.6084 (11)	0.5497 (12)	0.021 (5)*	
H1C	0.480 (4)	0.7033 (14)	0.5694 (12)	0.046 (6)*	
Cl1	0.95062 (9)	0.63979 (3)	0.44397 (3)	0.01993 (10)	

### Atomic displacement parameters (Å^2^)

**Table:**

	*U*^11^	*U*^22^	*U*^33^	*U*^12^	*U*^13^	*U*^23^
O1	0.0229 (7)	0.0200 (7)	0.0162 (7)	0.0064 (6)	0.0009 (6)	0.0022 (5)
O2	0.0319 (8)	0.0208 (7)	0.0125 (7)	0.0098 (6)	0.0014 (6)	0.0004 (6)
O3	0.0163 (6)	0.0236 (6)	0.0187 (7)	−0.0039 (5)	−0.0014 (6)	−0.0012 (6)
O4	0.0203 (6)	0.0207 (6)	0.0123 (7)	−0.0020 (5)	0.0007 (5)	0.0007 (5)
N1	0.0176 (7)	0.0152 (7)	0.0157 (7)	−0.0011 (8)	−0.0012 (8)	0.0011 (8)
C1	0.0241 (8)	0.0171 (8)	0.0130 (8)	−0.0048 (8)	−0.0022 (9)	0.0019 (8)
C2	0.0224 (9)	0.0175 (8)	0.0139 (9)	−0.0022 (7)	0.0019 (9)	−0.0013 (8)
C3	0.0158 (9)	0.0114 (8)	0.0169 (10)	−0.0020 (7)	0.0008 (8)	0.0009 (8)
C4	0.0197 (9)	0.0122 (8)	0.0125 (9)	−0.0017 (7)	−0.0013 (8)	0.0014 (7)
C5	0.0221 (10)	0.0114 (8)	0.0152 (9)	−0.0004 (7)	0.0016 (8)	−0.0025 (7)
C6	0.0162 (8)	0.0096 (7)	0.0180 (9)	−0.0041 (7)	−0.0017 (8)	−0.0002 (7)
C7	0.0174 (9)	0.0148 (8)	0.0191 (10)	−0.0023 (7)	−0.0032 (8)	0.0004 (8)
C8	0.0131 (9)	0.0147 (8)	0.0141 (10)	−0.0004 (7)	0.0006 (7)	0.0015 (7)
C9	0.0179 (9)	0.0089 (8)	0.0161 (9)	0.0023 (7)	0.0008 (8)	−0.0006 (7)
C10	0.0315 (12)	0.0255 (10)	0.0138 (10)	−0.0007 (9)	−0.0035 (9)	0.0022 (8)
Cl1	0.0227 (2)	0.0207 (2)	0.0164 (2)	0.00256 (19)	−0.0024 (2)	0.0008 (2)

### Geometric parameters (Å, º)

**Table:**

O1—C3	1.370 (2)	C2—H2A	0.9500
O1—H1	0.83 (2)	C3—C4	1.395 (2)
O2—C4	1.372 (2)	C4—C5	1.377 (2)
O2—H2	0.84 (2)	C5—C6	1.404 (2)
O3—C9	1.207 (2)	C5—H5	0.9500
O4—C9	1.326 (2)	C6—C7	1.517 (2)
O4—C10	1.458 (2)	C7—C8	1.539 (2)
N1—C8	1.494 (2)	C7—H7A	0.9900
N1—H1A	1.01 (2)	C7—H7B	0.9900
N1—H1B	0.92 (2)	C8—C9	1.515 (2)
N1—H1C	0.96 (2)	C8—H8	1.0000
C1—C6	1.390 (2)	C10—H10A	0.9800
C1—C2	1.390 (2)	C10—H10B	0.9800
C1—H1D	0.9500	C10—H10C	0.9800
C2—C3	1.385 (2)		
			
C3—O1—H1	110.8 (13)	C1—C6—C5	118.26 (16)
C4—O2—H2	110.8 (14)	C1—C6—C7	121.64 (15)
C9—O4—C10	116.42 (13)	C5—C6—C7	120.06 (15)
C8—N1—H1A	106.7 (12)	C6—C7—C8	115.09 (13)
C8—N1—H1B	116.0 (11)	C6—C7—H7A	108.5
H1A—N1—H1B	113.7 (16)	C8—C7—H7A	108.5
C8—N1—H1C	109.5 (12)	C6—C7—H7B	108.5
H1A—N1—H1C	100.5 (16)	C8—C7—H7B	108.5
H1B—N1—H1C	109.3 (16)	H7A—C7—H7B	107.5
C6—C1—C2	121.08 (17)	N1—C8—C9	108.29 (14)
C6—C1—H1D	119.5	N1—C8—C7	111.08 (14)
C2—C1—H1D	119.5	C9—C8—C7	112.92 (13)
C3—C2—C1	120.11 (17)	N1—C8—H8	108.1
C3—C2—H2A	119.9	C9—C8—H8	108.1
C1—C2—H2A	119.9	C7—C8—H8	108.1
O1—C3—C2	119.30 (16)	O3—C9—O4	125.64 (16)
O1—C3—C4	121.36 (15)	O3—C9—C8	124.63 (16)
C2—C3—C4	119.31 (16)	O4—C9—C8	109.72 (14)
O2—C4—C5	123.99 (16)	O4—C10—H10A	109.5
O2—C4—C3	115.46 (14)	O4—C10—H10B	109.5
C5—C4—C3	120.55 (16)	H10A—C10—H10B	109.5
C4—C5—C6	120.65 (16)	O4—C10—H10C	109.5
C4—C5—H5	119.7	H10A—C10—H10C	109.5
C6—C5—H5	119.7	H10B—C10—H10C	109.5
			
C6—C1—C2—C3	−1.7 (2)	C4—C5—C6—C7	179.30 (14)
C1—C2—C3—O1	179.68 (13)	C1—C6—C7—C8	−98.02 (19)
C1—C2—C3—C4	1.6 (2)	C5—C6—C7—C8	84.2 (2)
O1—C3—C4—O2	1.3 (2)	C6—C7—C8—N1	71.39 (19)
C2—C3—C4—O2	179.33 (14)	C6—C7—C8—C9	−50.5 (2)
O1—C3—C4—C5	−177.96 (14)	C10—O4—C9—O3	−3.4 (2)
C2—C3—C4—C5	0.1 (2)	C10—O4—C9—C8	175.40 (12)
O2—C4—C5—C6	179.21 (15)	N1—C8—C9—O3	−3.5 (2)
C3—C4—C5—C6	−1.6 (2)	C7—C8—C9—O3	119.97 (17)
C2—C1—C6—C5	0.2 (2)	N1—C8—C9—O4	177.71 (12)
C2—C1—C6—C7	−177.58 (14)	C7—C8—C9—O4	−58.84 (18)
C4—C5—C6—C1	1.5 (2)		

### Hydrogen-bond geometry (Å, º)

**Table:**

*D*—H···*A*	*D*—H	H···*A*	*D*···*A*	*D*—H···*A*
N1—H1*C*···Cl1^i^	0.96 (2)	2.29 (2)	3.209 (2)	161 (2)
N1—H1*B*···Cl1	0.92 (2)	2.49 (2)	3.178 (2)	132 (1)
N1—H1*A*···Cl1^ii^	1.01 (2)	2.13 (2)	3.112 (2)	163 (2)
O2—H2···Cl1^iii^	0.84 (2)	2.26 (2)	3.086 (2)	167 (2)
O1—H1···O2	0.83 (2)	2.27 (2)	2.698 (2)	113 (2)
O1—H1···O3^iv^	0.83 (2)	2.26 (2)	3.029 (2)	155 (2)
C8—H8···O3^ii^	1.00	2.44	3.300 (2)	144
C10—H10*C*···O1^v^	0.98	2.47	3.137 (3)	125

Symmetry codes: (i) *x*−1/2, −*y*+3/2, −*z*+1; (ii) *x*−1, *y*, *z*; (iii) −*x*+3/2, −*y*+1, *z*+1/2; (iv) −*x*+2, *y*−1/2, −*z*+3/2; (v) −*x*+2, *y*+1/2, −*z*+3/2.
